# SOCCER_Index_: An Estimate of Recreational Soccer Players’ Physical Ability by Health Status and Lifestyle Habits

**DOI:** 10.3390/sports14020068

**Published:** 2026-02-05

**Authors:** Beatrice De Lazzari, Federico Caramia, Filippo Lupi, Paolo Salvatore, Giuseppe Vannozzi, Valentina Camomilla

**Affiliations:** 1 Department of Human Movement and Health Science, University of Rome “Foro Italico”, 00135 Rome, Italy; beatrice.delazzari@uniroma4.it (B.D.L.); federico.caramia@uniroma4.it (F.C.); valentina.camomilla@uniroma4.it (V.C.); 2 Interuniversity Centre of Bioengineering of the Human Neuromusculoskeletal System, University of Rome “Foro Italico”, 00135 Rome, Italy; 3 GoSport s.r.l., 00191 Rome, Italy; f.lupi@gosport.tech (F.L.); p.salvatore@gosport.tech (P.S.)

**Keywords:** football, regression, amateur, performance assessment, fitness profile

## Abstract

Soccer is practiced by professionals, amateurs, and recreational players. The physical assessment tools used by professionals are rarely available in recreational settings. Given the widespread participation and potential health benefits of soccer activity, it becomes essential to identify simple and accessible indicators that can help to characterize physical ability in non-professional players. This cross-sectional observational work explores which health status and lifestyle indices can be useful to estimate physical ability in recreational male soccer players when field testing is not feasible. Sixty-six participants volunteered in the study. Five performance field tests were conducted, and a related overall physical ability index (KPI_tot_) was defined, while a questionnaire was developed to investigate nine BIO_Indices_ (BMI, age, physical activity level, job, alcohol consumption, smoking habits, sports career, occurring injuries, medical history). Data for the selected performance tests are reported for the recruited recreational athletes. KPI_tot_ was estimated from BIO_Indices_, using a stepwise backward regression. The selected model, named SOCCER_Index_, incorporates six out of nine BIO_Indices_, excluding smoking habits, sports career, and medical history (R^2^ = 0.536). In conclusion, with a simple questionnaire, an estimate of soccer players’ physical ability can be obtained. Further data collection is needed to obtain a more generalizable and robust SOCCER_Index_.

## 1. Introduction

Soccer is an intermittent sport practiced worldwide by both professional athletes and amateurs [1], characterized by repeated high-intensity efforts, sustained by mixed aerobic–anaerobic energy metabolism [1,2], and fatigue-related performance decrements [2,3]. Players’ levels and match intensity modulate this scenario, since intensity drops in lower leagues or with older players [1]: professionals run 28% more and do 58% more sprints than amateurs, who often substitute high-intensity effort with lower-intensity activity or rest due to less focus on physical training [4]. Players’ levels also reflect on the injury profile: amateurs experience about 3.2 injuries per 1000 match hours, with even higher incidence during training [5]. Absenteeism from games or training is reported at ~18% for professionals and ~23% for amateurs [6]. Direct epidemiological data on injury incidence in unorganized recreational soccer are scarce, due to the heterogeneity of playing contexts and the lack of structured surveillance systems. Nevertheless, injury risk in recreational players can be contextualized by cautious extrapolation from organized amateur settings, where evidence suggests that mismatches between physical preparation and match demands are associated with injury occurrence [5]. Their risk increases with older age, agonist/antagonist muscle imbalance, reduced joint range of motion, and abnormal BMI (e.g., overweight or obese) [5].

Physical capacities (e.g., agility, endurance, speed, power, …), underlying both performance potential and risk of injury, are evaluated through in-field testing, providing key performance indicators (KPIs). These tests are regularly performed in elite athletes and only rarely on amateurs [7], due to practical issues: limited time/space, low accessibility to physical testing facilities, and limited coaches’ knowledge. Although in recent years this gap has been reduced in amateur teams, such a difference persists in players not enrolled in a team or having a training-game routine.

Four soccer-specific physical fitness qualities are key in a recreational setting: capacity to sustain repeated high-intensity efforts (with short recovery), sprint and change-of-direction performance, intermittent aerobic fitness, and lower-limb strength and power. KPIs relative to these qualities can be assessed by widely used tests applicable to recreational and untrained individuals: the maximal Yo-Yo Intermittent Recovery Test Level 1 (Yo-Yo IR1), which evaluates intermittent endurance and recovery capacity and has been associated with players’ agility characteristics and aerobic capacity [8]; the Repeated Sprint Ability (RSA) which captures repeated sprint performance with change of direction ability and short recovery and is influenced by aerobic/anaerobic capacity [2,9]; the countermovement jump (CMJ) and standing long jump (SLJ), which provide complementary field assessments of lower-limb strength/power in vertical and horizontal directions [4,10]; and the 30 m Sprint test to assess near-maximal sprint capability over a distance longer than most high-intensity soccer sprints in recreational players [11].

Few studies analyzed the health-related physical fitness of male recreational players in soccer: untrained adolescents have been compared to amateur soccer players [12], and untrained young adults to sedentary and running-trained peers [13]. Limited emphasis on physical capacities evaluation parallels the fact that recreational players are exposed to important risks of injury, and with an absenteeism rate of 23% [6]. As reported by Junge et al. [14], better education regarding injury prevention strategies could reduce this trend.

General fitness profile assessment tools offer a broad evaluation of physical status; however, complementing this perspective with soccer-specific demands (e.g., intermittent running, changes of direction, and repeated high-intensity actions) may improve the contextual relevance of fitness screening in recreational soccer. This consideration motivated the development of a soccer-specific yet practical screening tool for recreational players, providing a performance-oriented context that relates health and fitness screening to the functional demands of soccer.

Overall, looking at the fitness profile, defined as a comprehensive assessment that provides an ideal starting point for any activity to be planned or a tool to check the progress in a training routine, there are biometric quantities (e.g., anthropometry, health status, lifestyle habits) that proved to be related to several aspects of the fitness profile:

- Body mass index (BMI) is a screening factor for health, correlated to quality of life [15]. Higher values correlate with lower sprinting, jumping, and anaerobic power [16];
- Age is a major risk factor for chronic degenerative diseases, according to WHO guidelines (2004) [17], a factor increasing soccer injury risk in both professional [6,18] and amateur players [5], and entailing a decline of physical activity [19];
- Alcohol [20] and cigarettes [21] consumption, major factors of health, have an adverse effect on cardiovascular fitness and heart rate response to exercise;
- The presence of chronic diseases can affect fitness [22];
- Previous injuries can affect a player’s health and fitness profile, increasing possible re-injury [18];
- Early engagement in sport and sustained habitual participation during youth may be linked to higher physical activity levels later in adulthood, while a prolonged sport career may facilitate the maintenance of an active lifestyle through adolescence and beyond [23,24]. Sport career history can thus be considered as a feasible proxy of long-term exposure to structured physical activity [25];
- While people who are physically active at work exhibit better health and fitness patterns [26], other factors (e.g., staying seated or maintaining poor posture for long periods of time) relative to players’ occupation have negative effects on health status [19];
- A high physical activity level has positive effects on physical fitness [27].

These domains, together, are feasible to capture through brief self-report items suitable for large-scale recreational settings. Although each factor is individually supported by prior evidence, they are rarely integrated into a single brief, self-administered tool tailored to recreational soccer, and it remains unclear whether a proper combination of these factors can reasonably predict players’ physical ability.

Accordingly, we developed a short questionnaire-based model (SOCCER_Index_) to estimate a composite field-test fitness index (KPI_tot_), defined as the average of the selected soccer-specific KPIs, that reflect corresponding core physical fitness capacities relevant to soccer performance. The work proceeds in three steps: (i) selection of questionnaire-derived proxies (BIO_Indices_) grounded in prior evidence; (ii) construction of a composite fitness reference (KPI_tot_) from standard field tests; and (iii) exploratory modelling of the relationship between BIO_Indices_ and KPI_tot_ in a sample of adult recreational male soccer players, within a cross-sectional and context-specific framework.

## 2. Materials and Methods

### 2.1. Study Design

**Figure 1:**
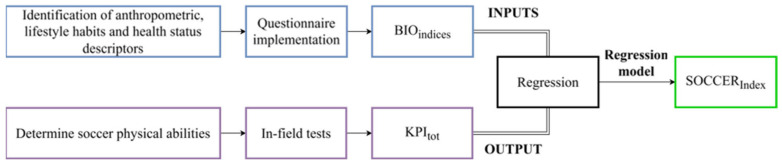
Workflow to estimate SOCCER_Index_. From the left, the identification of lifestyle indicators and physical assessment relevant for soccer; then, questionnaire implementation based on the fields of interest and selection of in-field tests. For each field of interest, a biometric indicator is evaluated, and for each test, a KPI is selected. Finally, the relation coming from the regression method that links BIO_Indices_ to the KPI_tot_ is called SOCCER_Index_.

The SOCCER_Index_ is an exploratory and predictive model defined through a regression analysis estimating players’ physical fitness, measured during in-field tests. Within the same time window, biometric quantities were determined through a questionnaire. More details are in Figure 1:

- Biometric quantities were selected to represent lifestyle indicators related to the player’s physical characteristics, and investigated via a closed-form questionnaire, whose results were grouped into BIO_Indices_, one per quantity.
- The principal aspects affecting players’ performance and their physical fitness were identified, and related in-field tests were performed to obtain specific KPIs, then combined in a single index named KPI_tot_.
- Regression analysis was performed using the questionnaire BIO_Indices_ as inputs and the KPI_tot_ as output. Thus, SOCCER_Index_ is defined following the relation that estimates KPI_tot_ starting from the obtained answers.

### 2.2. Questionnaire Design

To ensure ease of use, the questionnaire was administered via smartphone app and was developed based on scientific literature, adapting existing questionnaires to cover multiple domains. To maximize feasibility and completion in an app-based self-administered setting [28], respondent burden was minimized through a short, closed-form survey, prioritizing items with established links to physical fitness and health. The questionnaire investigated four domains:

1. Anthropometrics: subject’s BMI and age.
2. Health information: smoking habits, alcohol consumption, medical history, and injuries in the last 12 months.
3. Habits: sports career and work-related lifestyle.
4. Physical activity level: evaluated through the IPAQ-FS questionnaire [29].

The questionnaire was piloted in a convenience sample (*n* = 21) of recreational players to assess comprehension and completion time. Based on feedback, wording and response options were refined to reduce ambiguity. Median completion time was 5 min. Item wording, response options, and scoring rules are reported in Table 1 and Table 2. The final questionnaire, composed of 20 questions, investigated nine BIO_Indices_ as follows:

1. Body mass index score (BMI_Index_): BMI is calculated as body mass (kg)/height^2^ (m^2^). A percentage score is obtained (Table 1), accounting for “underweight” and “overweight” as conditions affecting body structure and daily living [30].
2. Age (Age_Index_): it evaluates players’ age by weighting age ranges based on their prevalence in professional soccer as a reference [31] (Table 1), to approximate the age window typically associated with peak physical performance, even if these ranges do not necessarily mirror the age distribution in recreational players.
3. Smoking habits (Smoke_Index_): participants are classified as smokers or non-smokers, and a percentage reduction is assigned to the former based on evidence that smoking reduces cardiovascular endurance by ~21% [32] (Table 1).
4. Alcohol consumption (Alcohol_Index_): alcohol consumption was assessed as the sum of the outcome scores of two questions adapted from the AUDIT questionnaire [33] that inquired about the daily amount and frequency of consumption (Table 1).
5. Medical history (Medical_Index_): it is assessed as the presence/absence of chronic diseases, referring to current/past pathologies that could affect sports activity. Diseases were characterized according to the Italian Cardiological Guidelines for Competitive Sport Eligibility [34], which allows athletes with a positive ECG evaluation to participate in competitive sports. This sports medical certification was used to divide the participants into three groups (Table 1): “healthy” with no chronic diseases, can play at a competitive level; “controlled chronic disease”, can do non-competitive activity under medical prescription; “severe chronic disease”, can do low-impact sports activity when prescribed.
6. Occurring injuries (Injury_Index_): this index was obtained from a single question on recent inactive days due to injury, with percentage scores decreasing with longer absence, reflecting severity (Table 2), considering as reference values the time spent for amateur and recreational players that typically do not follow a structured return-to-sport programme like professional athletes [6]. For this reason, even short rest periods are considered to categorize injury severity. Relapse risks can affect amateurs’ health status at a higher incidence than in athletes (16.6% and 15.4%, respectively), and are considered important also for recreational athletes [35]. The value of the Injury_Index_ was developed according to the type of injury, which was defined based on the number of days spent without training. Within these time-loss ranges, it was possible to classify injuries using medical criteria (e.g., rupture, strain). Injury categories were assigned pragmatic weights to obtain a simple, screening-oriented score; this mapping is heuristic and prioritizes feasibility over clinical grading. This represents an exploratory attempt to quantify injury severity with respect to inactivity days.
7. Sport career (Career_Index_): this index classified participants according to prior soccer-playing experience (ex-professional, ex-amateur, or none), with scores reported in Table 2.
8. Work-related indicator (Work_Index_): this was defined to account for participants’ physical activity at work and commuting modality, selected as relevant to the fitness profile; the highest scores were assigned to physically demanding jobs and active commuting [26] (Table 2).
9. Physical activity level (PAL_Index_): this index was defined to estimate the physical activity level, similar to the 12-item IPAQ-SF questionnaire [29]. Although practical and widely applied to investigate PA volume/intensity, it tends to overestimate activity when compared to devices such as pedometers, actometers, and accelerometers [14] and relies on the interviewer’s interpretation of open responses to compute MET (Metabolic Equivalent of Task); this can lead to potential overestimation/underestimation of PA [29]. To improve accuracy and allow self-administration, two adaptations were applied:

- Open responses were replaced with closed ranges of days/hours, providing options closer to users’ lifestyles.
- MET values were not calculated, reducing overestimation/underestimation risks.

In line with IPAQ-SF guidelines, physical activities are divided into vigorous, moderate, and walking categories, and participants are classified as very active, active, or inactive as in the IPAQ-SF21 (Table 2).

**Table 1:** Body Mass Index score section—BMI values and relevant BMI levels are transformed into a percentage score, BMI_Index_, as described in the “Relation” column. Age index section—In the first column, age in years; the second column reports the age ranges; in the third column, the Age_Index_ as percentage scores associated with age; in the fourth column, the relation between age and Age_Index_. Smoking habits section—Smoking status, the percentage value associated with the response, and the relation between the response and the associated percentage value are reported. Alcohol consumption section—Answers to the “frequency of alcohol consumption” question (1), and related scores associated with the answers. In the third column, the answers to the “amount of alcohol in a day” question (2) and the corresponding score in the fourth question are provided. Scores are taken from the AUDIT questionnaire [33]. In the remaining columns, the total score, the corresponding percentage, and the relationship criterion between the total score and the Alcohol_Index_ are reported. Medical history section—Answers, examples, and corresponding Medical_Index_ percentage values related to medical history in the first three columns. The fourth column shows the related general criterion.

**Body Mass Index Score**						
**BMI Value [kg/m^2^]**	**BMI Levels**	**BMI** **_Index_**	**Relation**			
**<15**	Underweight	45%	Fixed percentage value			
**15–17.9**	Underweight	46–75%	For every BMI increase of 0.1, BMI_Index_ increases of 1% BMI_Index_ = 45% + (BMI − 14.9) × 1%			
**18–22**	Normal	76–100%	For every BMI increase of 0.1, BMI_Index_ increases of 1% BMI_Index_ = 75% + (BMI − 17.9) × 1%			
**22.1–24.9**	Normal	99–71%	For every BMI increase of 0.1, BMI_Index_ decreases of 1% BMI_Index_ = 100% − (BMI − 22) × 1%			
**25–30**	Overweight	70–20%	For every BMI increase of 0.1, BMI_Index_ decreases of 1% BMI_Index_ = 71% − (BMI − 24.9) × 1%			
**30.1**	Obese	19%	Fixed percentage value			
**30.2–32.6**	Obese	18–6%	For every BMI increase of 0.2, BMI_Index_ decreases of 1% BMI_Index_ = 19% − (BMI − 30) × 1%			
**32.7–33.8**	Obese	5%	Fixed percentage value			
**34–34.9**	Obese	0%	Fixed percentage value			
**>35**	Extremely obese	0%	Fixed percentage value			
**Age**						
**Age (years)**	**Age range**	**Age** **_Index_**	**Relation**			
**14–17**	Adolescent	60–80%	For every 1-year increase, there is an increase of 5% Age_Index_ = 60% + (age − 13) × 5%			
**18–27**	Young adult	82–100%	For every 1-year increase, there is an increase of 2% Age_Index_ = 80% + (age − 17) × 2%			
**28–36**	Adult	98–74%	For every 1-year increase, there is a decrease of 2% Age_Index_ = 100% − (age − 27) × 2%			
**37–51**	Senior	72–44%				
**52–73**	Over	42–0%				
**Smoking habits**						
**Smoker**	**Smoke_Index_**	**Relation**				
**No**	100%	Fixed value associated with non-smokers’ cardiovascular endurance				
**Yes**	79%	Percentage value associated with smokers’ cardiovascular endurance (decrease of 21%, following Jeon [32])				
**Alcohol consumption**						
**Frequency of alcohol consumption**	**Score**	**Amount of alcohol x day**	**Score**	**Total score**	**Alcohol_Index_**	**Relation**
**Never**	0	1–2	0	0	100%	Percentage values associated with the total score obtained by summing the answers to questions related to alcohol consumption
**Once a month**	1	3–4	1	1–2	75%	
**Two to four times a month**	2	5–6	2	3–4	50%	
**Twice or three times a week**	3	7–9	3	5–6	25%	
**>four times a week**	4	≥10	4	7–8	0%	
**Medical history**						
**Total score**	**Examples**		**Medical_Index_**		**Relation**	
**No chronic** **diseases**	-		100%		-	
**Controlled chronic diseases**	Hypertension, diabetes, and endocrine system problems		50%		The ones controlled through drugs and that do not expose the athlete to risks for physical activity.	
**Severe chronic** **diseases**	Severe problems with the spine or locomotor apparatus or oncological pathologies belong to this category		0%		Not controlled through pharmacological solutions, or that can limit the subject’s physical capacity.	

**Table 2:** “Occurring injuries” section—Days at rest due to an injury and corresponding gravity level. In the third column, the percentage values are associated with each answer. In the fourth column, the relation. “Sports career” section—In the first column, the previous soccer experience: ex-professional player, ex-amateur player, or without experience. In the second column, the corresponding percentage values are shown. “Work” section—In the first and third columns, there are two questions regarding the type of job and the type of transport to reach it. In the second and fourth columns, the corresponding scores; in the fifth column, the final score obtained as the sum of the two answers, along with the corresponding percentage value [26]. “Type of activities” and “Physical activity level” sections—In the first column, the type of activities investigated through the 12 questions of the IPAQ-SF; in the second column, the examples associated with them. Physical activity level outcomes from the IPAQ-FS, descriptions of the outcomes, and the associated percentage values are in the first, second, and third columns, respectively.

**Occurring Injuries**						
**Days at rest due to injury**		**Gravity level**		**Injury** **_Index_**	**Relation**	
**0**		No gravity		100%	Percentage values associated with the days at rest due to the injury.	
**2–4**		Slight gravity		75%		
**5–10**		Lower gravity		55%		
**11–15**		Middle-lower gravity		25%		
**16–30**		Middle gravity		15%		
**≥31**		Higher gravity		0%		
**Sports career**						
**Previous football experience**				**Career** **_Index_**		
**Ex Pro**				**100%**		
**Ex Amateur**				**50%**		
**Work**						
**1. Type of work**	**Score**	**2. How to go to work**	**Score**	**Final score**	**Work** **_Index_**	**Relation**
**Call centre**	0	Foot or bicycle	3	7	100%	The percentage value obtained after summing the scores of the two questions 1 and 2
**Employee**						
**Smart worker**						
**Waiter**	1					
**Student**				5	75%	
**Teacher**		Public transport	1			
**Trader**				4	60%	
**Clerk**						
**Rider**	2			3	45%	
**Healthcare personnel**						
**Trainer**		Car or scooter	0	2	30%	
**Police**						
**Agricultural work**	4			1	15%	
**Construction company**						
**Forestry work**				0	0%	
**Type of activities**						
**Type of Activity**				**Examples**		
**Vigorous**				Weightlifting, high-intensity aerobic activity, and cycling		
**Moderate**				Carry light loads, ride a bicycle at a regular pace, exercise, and jog		
**Walk**				If the activity results only in walking constantly for 10–15 min during the day		
**Physical Activity Level**						
**Physical Activity Level categories**	**Description**					**PAL** **_Index_**
**Very active**	The subject must engage in vigorous physical activity more than three times a week or must engage in 7 or more days of any combination of walking, moderate intensity, or vigorous intensity activities.					100%
**Active**	The subject must engage in 3 or more days of vigorous intensity activity and/or walking of at least 30 min per day; or 5 or more days of moderate intensity activity and/or walking of at least 30 min per day; or 5 or more days of any combination of walking, moderate intensity, or vigorous intensity activities.					50%
**Inactive**	The subject must not engage in vigorous physical activity more than three times a week, must not do vigorous moderate more than 5 days a week.					0%

### 2.3. In-Field Tests: Selection and Protocol

To assess soccer-specific key physical fitness qualities, five validated in-field tests were selected to characterize:

- capacity to recover from repeated high-intensity sprints—Yo-Yo Intermittent Recovery Test Level 1 (Yo-Yo IR1);
- sprint and change directions abilities—repeated sprint ability test (RSA);
- aerobic and recovery capacities—30 m sprint ability test;
- lower limb strength and power—countermovement jump (CMJ) and standing long jump (SLJ).

**Figure 2:**
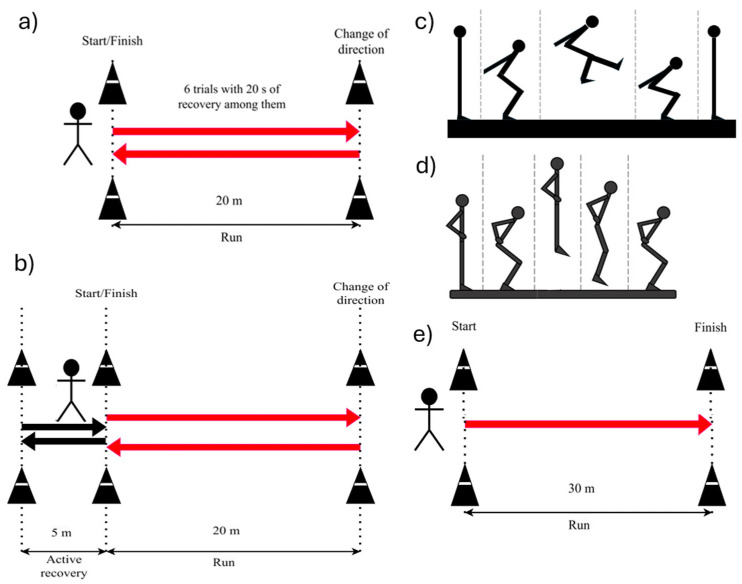
Schemes for the implemented tests: (**a**) Yo-Yo Intermittent Recovery Test Level 1 (Yo-Yo IR1); (**b**) Repeated Sprint Ability (RSA); (**c**) Countermovement Jump (CMJ) vertical execution (the frames refer to the same position in space); (**d**) Standing-Long Jump (SLJ) execution; (**e**) 30 m sprint scheme.

Tests were chosen based on feasibility in recreational settings and widespread use in soccer literature. For each test, a specific percentage KPI was computed, and their average was assumed to represent the overall physical ability (KPI_tot_). The description of each test and corresponding KPI is given below; their graphical representation is included in Figure 2.

The questionnaire and tests were administered separately, without prior explanation of the final aim, to avoid influencing the answers. Physical tests were performed over two days, to limit physical fatigue: Yo-Yo IR1 and RSA test on different days, others were distributed randomly.

#### 2.3.1. Yo-Yo Intermittent Recovery Test Level 1 (Yo-Yo IR1)

Participants run 20 m shuttles, being in time with time intervals decreasing at each round and marked by a beep sound, interspersed with 10 s of active recovery (walking or slow running in a 5 m space). KPI_YoYo_ is the total distance run until the time constraints are not met twice or until quitting.

#### 2.3.2. Repeated Sprint Ability (RSA)

Participants perform six shuttle runs of 20 + 20 m with 20 s recovery, aiming at exploring change of direction ability and sprint recovery. KPI_RSA_ is defined as the RSA_decr_ in Rampinini et al. [9]:

RSA_decr_ (%) = [100 ∗ (RSA_mean_/RSA_best_)] − 100 (1)

where RSA_mean_ is the mean value of the six-time intervals used to do each shuttle, and RSA_best_ is the shortest time among the six shuttles.

#### 2.3.3. Jumping Assessment

Lower-limb strength and power were evaluated with countermovement jump (CMJ) and standing long jump (SLJ). These jump tests were included due to the following reasons: CMJ reflects vertical power and stretch–shortening cycle utilization, while SLJ captures horizontal power expressed.

Participants start CMJs with their hands on their hips; at the start, suddenly bend their knees and, without stopping the movement, jump upwards. KPI_CMJ_ is the jump maximal height, out of three jumps, measured by an inertial sensor [36] (Gyko, Microgate, Bolzano, Italy, fs = 1000 samples/s; full scale range: ±16 g, ±2000 deg/s), and expressed as a percentage relative to professional soccer reference (46 cm [37]).

The SLJ is performed with the participant in a standing position with their hands on their hips and, at the start, performing a forward jump with both limbs and landing on the ground with their feet close together. KPI_SLJ_ is the longest jumped length, out of three jumps, expressed in percentage of professional soccer reference (280 cm [38]) using a piecewise linear interpolation. KPI_Jumps_ is calculated as the average of KPI_CMJ_ and KPI_SLJ_.

#### 2.3.4. The 30-m Sprint Test

The 30 m sprint test assesses acceleration and near-maximal sprint performance over a 30 m distance. KPI_Sprint_ is the time spent in a 30-m sprint, expressed as a percentage value with respect to the elite reference [11] using piecewise linear interpolation.

#### 2.3.5. Final KPI (KPI_tot_)

For the selected tests, the identified KPIs correspond to percentage values obtained, except for CMJ, using Microsoft^®^ Excel^®^ for Microsoft 365 MSO (Version 2501) using piecewise linear interpolation, across various levels corresponding to different soccer categories, as shown in Table 3. Percentage KPIs were obtained by mapping each test outcome to literature-based reference ranges (Table 3), where 100% corresponds to the top (professional/elite) reference values reported in the cited studies for each test.

KPI_tot_ is the final KPI index obtained by averaging the KPIs coming from the performed tests, after combining the jump ones:

KPI_tot_ = (KPI_YoYo_ + KPI_RSA_ + KPI_Jumps_ + KPI_Sprint_)/4 (2)

KPI_Jumps_ = (KPI_CMJ_ + KPI_SLJ_)/2 (3)

### 2.4. Participants

Sixty-six male recreational participants (age: 31 ± 11 years, mass: 76 ± 9 kg, stature: 179 ± 6 cm) volunteered after giving informed consent. The study was approved by the local Internal Review Board (CAR 94/2021/Rev2022/Rev2023). Only physically active, healthy recreational soccer players were included, excluding individuals who underwent either lower limb surgery or injury in the six months prior to the experimental session. Participants were recruited through local advertising and flyer distribution. As the study targeted recreational players, they were not affiliated with football clubs at the time of recruitment. As they are not registered with a club, participants took the tests both in season and off-season. Participants completed the questionnaire and performed the in-field tests. Before the testing session, each participant completed a 15-min warm-up consisting of 5 min of low-intensity jogging, mobility exercises, bodyweight exercises (e.g., half squats, lunges), and low-intensity shuttle runs.

### 2.5. Statistical Analysis and Soccer Index Modelling (SOCCER_Index_)

Statistical analyses were performed using IBM SPSS Statistics (version 28). Participants’ test-specific KPIs were mapped to percentage scores using the predefined reference ranges (Table 3). The distribution of participants across the corresponding percentage ranges was then calculated for each test to provide a descriptive profile of recreational players’ fitness.

For SOCCER_Index_ modelling, questionnaire-derived indicators (BIO_Indices_) were used as predictors in multiple linear regression inputs and test physical fitness score (KPI_tot_) as the dependent variable. Given the exploratory nature of the study and the limited sample size, a backward stepwise linear regression approach was adopted as a pragmatic variable reduction strategy to estimate the individual contribution of each BIO_Indices_ to KPI_tot_. The full model initially included all BIO_Indices_; progressively more parsimonious models were then iteratively created by a step-by-step removal of the less significant index. For each regression model, R^2^ and adjusted R^2^ (R^2^_adj_) values were obtained, describing the correlation between BIO_Indices_ and KPI_tot_ for the indices contributing to the latter. The final model was selected based on the best trade-off between R^2^ and R^2^_adj_. The regression equation corresponding to the selected model was named SOCCER_Index_ and used as a reference for the evaluation of the player’s fitness.

This same modelling procedure was also performed using single-test KPIs as outcome, to explore associations between BIO_Indices_ and specific fitness components.

Correlation strength was classified as weak (R^2^ < 0.3), moderate (0.4 < R^2^ < 0.7), and strong (R^2^ > 0.7) [39]. Analyses were performed using IBM SPSS 28.

## 3. Results

Table 4 reports the test results for the analyzed group. Most participants were classified as active/recreational in Yo-Yo IR1 and RSA tests, but as amateur or semi-pro for SLJ and 30 m sprint test (amateurs: 30 participants for SLJ, 23 for 30 m test; semi-pro: 17 for SLJ, 19 for 30 m test), with four participants reaching professional level for CMJ [37].

The models estimating KPI_tot_ through backward regression analyses, for each decreasing number of variables, are reported in Table 5. The best KPI_tot_ model, which allows the estimation of the SOCCER_Index_, incorporates six BIO_Indices_ (BMI_Index_, Age_Index_, PAL_Index_, Injury_Index_, Alcohol_Index_, Work_Index_) explaining 53.6% of the variance in overall physical ability (KPI_tot_, R^2^_adj_ = 0.496—Table 5).

The best models with at least a moderate correlation and selected for each specific KPI (KPI_Sprint_, KPI_Yo-Yo_, KPI_SLJ_, KPI_CMJ_) are summarized in Table 5. KPI_RSA_ presented a weak correlation with the selected BIO_Indices_. Detailed tables reporting backward regression analyses for all KPIs are provided in Appendix A.

## 4. Discussion

This study provides evidence that selected lifestyle factors and health status indices can serve as indicators of physical ability in recreational male soccer players. The model explained 53.6% of the variance in KPI_tot_ within the studied sample, supporting its exploratory potential. This finding has important practical implications, as it demonstrates that a simple questionnaire can provide meaningful insights into recreational players’ physical abilities when in-field tests are not feasible. By combining specific physical abilities with more general factors (e.g., job, medical history), the model offers a comprehensive fitness profile related to soccer capacities (e.g., aerobic capacity, lower limb strength) that could be useful to guide the development of specific training planning. While SOCCER_Index_ provides insights into recreational players’ physical abilities, it is not intended to replace medical clearance, which remains essential for safe participation and individualized training prescription. Furthermore, it could be a more specific soccer screening tool that respects other assessments [29] and a low-cost solution compared to expensive technologies that serve a similar aim [40].

Our participant group’s heterogeneity in test performance—ranging from recreational to semi-professional levels across different assessments (Table 4)—reflects the diverse nature of recreational soccer players, highlighting the complex interplay between lifestyle factors, training history, and physical performance in this population. Participants’ heterogeneity reflects different lifestyle habits, health status, and physical capacities (e.g., lower limb strength, velocity, aerobic capacity), which could not be controlled in this observational design, limiting direct comparison with other studies that propose a longitudinal approach with controlled training sessions [13].

BIO_Indices_ are simplified descriptors of complex constructs, whose interaction with soccer performance and health status has been proven in the literature with more distinctive quantitative/qualitative data than those provided by our simple questions. Grouped together, they contribute to the explanation of the SOCCER_Index_, building upon known influences: BMI, age, and previous injuries influence soccer performance [4,15]; alcohol can compromise motor skills and reactive capacity [41]; work-related indicators and habitual PA directly impact physical condition [29].

These BIO_Indices_ are indicators of specific KPIs (Table 5):

- The BMI score (increasing for BMI values up to 22 and then decreasing for higher BMI values) and age score (increasing up to 27 years and then decreasing) are positively correlated with all KPIs except KPI_RSA_, which was not well predicted.
- Physical activity level is a recurrent indicator in different tests: being active is associated with higher aerobic/anaerobic capacity (KPI_Yo-Yo_), sprint performance (KPI_Sprint_), and lower limb strength, particularly in the horizontal component (KPI_SLJ_).
- Limited alcohol consumption and work-related indicators are positively correlated with aerobic/anaerobic capacity and sprint performance. These indicators do not influence jumping performance.
- The “occurring injuries” indicator is connected to jump performance, but not with sprint performance and aerobic/anaerobic capacity.

Among the BIO_Indices_ that did not enter the SOCCER_Index_ model, we find smoking habits, sports career, and chronic diseases. This may be due to the limited sensitivity of the self-reported categories and restriction of range in our cohort; future studies using more granular assessments and larger samples should re-evaluate their contribution, particularly for specific fitness components. Moreover, these descriptors present some correlations with single tests (Table 5):

- Smoking habits are correlated only with aerobic/anaerobic capacity.
- The sports career is correlated with the aerobic/anaerobic capacity and with vertical jump performance.
- The presence of chronic diseases is correlated with jump performance.
- In the following subsections, the presence/absence of correlation between all BIO_Indices_ and the overall physical fitness is explored in detail, discussing potential explanations.

### 4.1. SOCCER_Index_

Some health-relevant factors, such as smoking habits, medical history, and past sports career, despite their proven relevance to fitness, did not enter in the final multivariable SOCCER_Index_ model. This was likely due to the coarse measurement of our questionnaire: smoking consumption was categorized only as a yes/no habit, without investigating consumption; medical history was not differentiated across pathologies with greater or lower impact on performance. Moreover, there is limited variability in this factor within our convenience sample of healthy recreational players. Including more comprehensive information on these factors and enlarging the participating cohort could improve the questionnaire. Finally, previous sports experience may contribute less to a multivariable model of amateur players’ physical fitness. A more detailed investigation into a sport career (e.g., duration, category, intensity, and type of sport) could be included in the questionnaire to relate it to the in-field test.

### 4.2. 30-m Test

Sprint performance was explained with moderate correlations by the same BIO_Indices_ included in the SOCCER_Index_ estimation, except the Injury_Index_ (R^2^ = 0.417, Table 5). This absence suggests that inactive days may not strongly influence sprint performance. A more detailed categorization of injury types in the questionnaire could help to identify links with sprinting ability. However, future refinements should balance increasing details with ease of use of the questionnaire and the user’s time.

### 4.3. Yo-Yo IR1 Test

The KPI_Yo-Yo_ was estimated with moderate correlation using seven BIO_Indices_ (R^2^ = 0.550, Table 5), five of which are common with SOCCER_Index_: age, BMI, alcohol consumption, physical activity level, and work-related indicator. Physical activity and work-related activity showed particularly strong effects in the KPI_Yo-Yo_ model with respect to the SOCCER_Index_ one. Injury_Index_ is replaced by the Smoke_Index_ as a risk factor for the health status, reflecting the great aerobic/anaerobic demand of this test: a smoker can exhibit reduced respiratory and recovery capacity [32]. Although ex-athletes are expected to have better aerobic/anaerobic capacity [14] than beginners, sports career showed a negative association with KPI _Yo-Yo_ in this sample, which may reflect confounding by age decline, injury history, or other physiological components. A deeper analysis of sport type and career length could clarify this correlation.

### 4.4. SLJ and CMJ Tests

KPI_SLJ_ and KPICMJ models both showed moderate results, the former slightly weaker (Table 5, R^2^ < 0.4, and R^2^ = 0.431, respectively). Three out of six indicators were shared with SOCCER_Index_ in both models: age, BMI, and Injury_Index_ indicators. KPI_CMJ_ could also be associated with physical activity level, while KPI_SLJ_ could not.

Beyond the overlap with the general model, both jump models were associated with Career_Index_ and Medical_Index_. A sports career increases the chances of having a correct jumping technique, while the presence of certain pathologies could affect lower limb strength [22]. Even non-severe chronic conditions may impair muscle strength, thus impacting jumping performance.

### 4.5. RSA Test

Weak R^2^ values were obtained for all models (R^2^ < 0.2, Table 5), meaning that selected BIO_Indices_ were insufficient to describe RSA. Future studies should analyze additional descriptors or refine current questions.

Given the limited predictability of RSA from questionnaire data, an alternative version of KPI_tot_, excluding this component, was also evaluated and showed comparable model behaviour. RSA was therefore retained in the composite index due to its relevance for soccer-specific fitness.

### 4.6. Limitations and Future Development

A first limitation is the sample size (n = 66), which limits the analyses to exploratory regression modelling. Given the limited number of cases relative to the number of predictors, validation techniques were not applied. This constraint limits the robustness of the model (increasing the risk of overfitting) and the generalizability of the findings. The heterogeneity of the sample reflects the intended target population of recreational and sub-elite players for whom field testing is often impractical. However, this dataset size is not sufficiently large to describe the sub-groups, limiting statistical power. Finally, formal test–retest reliability or internal consistency analyses were not performed during piloting and represent a methodological limitation.

The use of the Italian soccer federation’s categorization may limit direct applicability to other countries. In particular, “professional athletes” play in the 1st to 3rd division, “semi-pro” play between the 4th and 5th division, “amateurs” between the 6th and 9th division, and “recreational players” play without belonging to a team. These classifications reflect the Italian competitive structure and may not be directly transferable to other national or organizational contexts. Where reference data were unavailable, values derived from other intermittent sports, such as basketball and handball [42,43], were used, which may introduce comparative biases.

To keep the survey concise, some questionnaire areas lacked detail. More specific questions, related to excluded BIO_indices_ (e.g., smoking quantities, medical condition type, injury recovery, sport career duration, and sport type), could help in verifying their contribution to the estimation of SOCCER_Index_, potentially enhancing their explanatory power. Furthermore, the scores associated with BIO_indices_, not completely defined by the literature, may introduce misclassification bias. The use of self-reported data to predict objective performance measures represents a limitation of the questionnaire approach, due to potential measurement error, social biases, and uncontrolled confounding factors (e.g., technical and tactical skills). In particular, participants with greater familiarity with training concepts may have provided responses that better reflect perceived rather than actual physical ability. These factors, together with the KPI_tot_ definition, may have contributed to unexplained variance and limited the applicability of SOCCER_Index_ for individual-level assessment.

Despite this, the approach is promising for soccer and potentially adaptable to other sports, populations (e.g., women or children), or different contexts (e.g., country, competitive level, training culture), with population-specific adjustments to testing protocols, reference standards, assessment of population-based biometric indices (e.g., growth factor or other physiological aspects), and validation.

Finally, the development of a smartphone application, incorporating the SOCCER_Index_, has a potential long-term perspective pending rigorous validation and reproducibility analyses. In this future context, an app-based implementation could provide a simple and convenient way to monitor physical ability in recreational players without access to professional assessment resources, democratizing access to evidence-based fitness evaluation for recreational athletes, and incorporating educational content about lifestyle, encouraging healthy behavioural changes among users.

## 5. Conclusions

This study introduces the SOCCER_Index_ as a cross-sectional, accessible, and exploratory tool for profiling physical ability in recreational male soccer players based on self-reported data. The findings indicate that selected lifestyle factors and health status indices are moderately associated with performance measures of physical ability, explaining approximately half of the observed variance.

Based on a simple questionnaire, the SOCCER_Index_ should provide a practical screening complement when in-field testing is not feasible. Its potential utility lies in the preliminary characterization of recreational players and the identification of general fitness patterns when direct physical testing is impractical.

Furthermore, the approach can be adapted and scaled for different sports and populations by tailoring in-field testing and questionnaire components. Longitudinal studies linking changes in BIO_Indices_ with performance improvements would clarify causal relationships underlying our model and the use of SOCCER_Index_ as a monitoring tool for tracking progress over time. Implementation within a smartphone application could further enhance accessibility and scalability across sports and athlete categories.

## Figures and Tables

**Table 3:** List of the categories created with reference to the ranges of the different tests.

Category	Yo-Yo IR1 [8]	RSA [2]	SLJ [10]	30 m [11]	CMJ [37]
**No active/no player**	<900 m	>10%	<151 cm	>6.5 s	Values referred to the jump of a professional player
**Active**	[900, 1100] m	[7%, 10%]	[151, 181] cm	[5.5, 6.7] s	
**Recreational**	[1100, 1500] m	[6%, 7%]	[181, 201] cm	[4.9, 5.5] s	
**Amateur**	[1500, 2000] m	[5.5%, 6%]	[201, 246] cm	[4.5, 4.9] s	
**Semi-pro**	[2000, 2800] m	[4.5%, 5.5%]	[246, 280] cm	[4, 4.5] s	
**Professional**	≥2800 m	<4.5%	≥280 cm [38]	≤4 s	≥46 cm

**Table 4:** Statistical results of the tests. For each category, the reference range (Range) and the number of participants (*N*) whose results fall within the corresponding range are provided. Moreover, the group’s mean and standard deviation were reported for each test. For RSA, the mean and standard deviation of each shuttle’s time were reported as well. In brackets, use round brackets if the upper or lower limit is excluded, and square brackets if it is included.

Category	Yo-Yo IR1 [8]		RSA [2]		SLJ [10]		30 m [11]		CMJ [37]	
	Range	*N*	Range	*N*	Range	*N*	Range	*N*	Range	*N*
**No active/no player**	<900 m	37	>10%	10	<151 cm	0	>6.5 s	0	Values referred to the jump of a professional player	
**Active**	[900, 1100] m	8	(7%, 10%]	16	[151, 181] cm	8	(5.5, 6.5] s	3		
**Recreational**	[1100, 1500] m	16	(6%, 7%]	7	[181, 201] cm	10	(4.9, 5.5] s	20		
**Amateur**	[1500, 2000] m	4	(5.5%, 6%]	7	[201, 246] cm	30	(4.5, 4.9] s	23		
**Semi-pro**	[2000, 2800] m	1	[4.5%, 5.5%]	10	[246, 280] cm	17	(4, 4.5] s	19		
**Professional**	≥2800 m	0	<4.5%	16	≥280 cm [38]	1	≤4 s	1	≥46 cm	4
	871 ± 451 m		6.6 ± 3.3%	222 ± 34 cm			4.7 ± 0.4 s	35 ± 6 cm		
**RSA shuttle time**	First		8.12 ± 0.93 s							
	Second		8.13 ± 0.82 s							
	Third		8.18 ± 0.80 s							
	Fourth		8.41 ± 0.87 s							
	Fifth		8.44 ± 0.86 s							
	Sixth		8.45 ± 0.83 s							

**Table 5:** “SOCCER_Index_ estimation” section—Models obtained through backward regression, considering the nine indicators of the questionnaire as inputs and KPI_tot_ as output. In the columns related to the model are reported: model number; R^2^, adjusted R^2^ (R^2^_adj_); and standard error of the estimates (SEE). In the subsequent columns, the constant value for each of the presented models and the BIO_Indices_ with their coefficients are listed. The selected model to define SOCCER_Index_ is indicated in light grey and shows which biometric indicators are most meaningful to estimate KPI_tot_. “Most meaningful models” section—Models for which selected biometric indicators of the questionnaire that are most meaningful to estimate KPI_Sprint_, KPI_Yo-Yo_, KPI_SLJ_, KPI_CMJ_, KPI_RSA_ are reported in comparison to the best model for SOCCER_Index_. The columns related to the model reported the following: KPI; R^2^, adjusted R^2^ (R^2^_adj_); standard error of the estimates (SEE), and β = constant value of the regression model. In the subsequent columns, the constant value for each of the presented models and the BIO_Indices_ with their coefficients are listed. General legend: BMI_Index_ = body mass index; Medical_Index_ = medical history index; PAL_Index_ = physical activity level index; Age_Index_ = age index; Injury_Index_ = occurring injuries index; Career_Index_ = sports career index; Alcoho_Index_ = alcohol consumption index; Work_Index_ = work-related index; Smoke_Index_ = smoke habits index; β = Constant value of the regression model.

**SOCCER_Index_ Estimation**													
**SOCCER_Index_ Model Estimates**				**BIO_Indices_**									
**Model**	**R^2^**	**R^2^_adj_**	**SEE**	**Β**	**BMI_Index_**	**Age_Index_**	**PAL_Index_**	**Injury_Index_**	**Alcohol_Index_**	**Work_Index_**	**Smoke_Index_**	**Medical_Index_**	**Career_Index_**
1	0.548	0.475	9.68	6.946	0.403	0.174	0.039	−0.084	0.133	0.055	0.089	0.025	−0.002
2	0.548	0.484	9.59	6.954	0.403	0.175	0.039	−0.084	0.132	0.055	0.089	0.025	-
3	0.546	0.491	9.53	9.621	0.402	0.173	0.038	−0.076	0.133	0.054	0.079	-	-
4	0.543	0.496	9.48	16.805	0.401	0.168	0.041	−0.075	0.139	0.059	-	-	-
5	0.536	0.497	9.47	18.327	0.396	0.178	0.042	−0.079	0.130	-	-	-	-
6	0.519	0.488	9.56	26.480	0.398	0.184	0.045	−0.075	-	-	-	-	-
7	0.498	0.474	9.69	20.979	0.384	0.194	0.040	-	-	-	-	-	-
8	0.481	0.464	9.78	22.145	0.387	0.203	-	-	-	-	-	-	-
**Most meaningful models**													
**KPI**	**R^2^**	**R^2^_adj_**	**SEE**	**Β**	**BMI_Index_**	**Age_Index_**	**PAL_Index_**	**Injury_Index_**	**Alcohol_Index_**	**Work_Index_**	**Career_Index_**	**Medical_Index_**	**Smoke_Index_**
SOCCER_Index_	0.543	0.496	9.48	16.805	0.401	0.168	0.041	−0.075	0.139	0.059	-	-	-
KPI_Sprint_	0.417	0.368	9.56	26.692	0.283	0.145	0.023	-	0.205	0.073	-	-	-
KPI_Yo-Yo_	0.550	0.496	13.37	−39.479	0.438	0.152	0.161	-	0.177	0.198	−0.193	-	0.219
KPI_SLJ_	0.379	0.316	12.12	44.637	0.213	0.235	0.069	−0.096	-	-	0.150	−0.093	-
KPI_CMJ_	0.431	0.384	11.18	53.438	0.230	0.219	-	−0.181	-	-	0.187	−0.109	-

## Data Availability

The data presented in this study are available on request from the corresponding author. Reasons for restrictions: the option for sharing data with third parties was not included in the consent form signed by the participants at the time of data collection.

## Appendix A

The models selected for each specific KPI (KPI_Sprint_, KPI_Yo-Yo_, KPI_SLJ_, KPI_CMJ_, KPI_RSA_).

**Table A1:** Models for which selected biometric indicators of the questionnaire are most meaningful to estimate KPI_Sprint_, KPI_Yo-Yo_, KPI_SLJ_, KPI_CMJ_, KPI_RSA_ are reported in comparison to the best model for SOCCER_Index_. In the columns related to the model are reported: KPI; R^2^, adjusted R^2^ (R^2^_adj_); standard error of the estimates (SEE), and β = constant value of the regression model. In the subsequent columns, the constant value for each of the presented models and the BIO_Indices_ with their coefficients are listed. Legend: BMI_Index_ = body mass index; Age_Index_ = age index; PAL_Index_ = physical activity level index; Injury_Index_ = occurring injuries index; Alcohol_Index_ = alcohol consumption index; Work_Index_ = work-related index; Career_Index_ = sports career index; Medical_Index_ = medical history index; Smoke_Index_ = smoke habits index.

Model Statistics for KPI_Sprint_				BIO_indices_									
Model	R^2^	R^2^_adj_	SEE	Β	BMI_Index_	Age_Index_	Alcohol_Index_	Work_Index_	PAL_Index_	Career_Index_	Smoke_Index_	Injury_Index_	Medical_Index_
1	0.434	0.343	9.74	18.237	0.287	0.146	0.223	0.076	0.025	−0.049	0.111	−0.039	0.026
2	0.432	0.352	9.68	20.932	0.286	0.145	0.224	0.075	0.025	−0.047	0.100	−0.031	-
3	0.427	0.358	9.63	19.087	0.280	0.148	0.225	0.080	0.023	−0.056	0.100	-	-
4	0.421	0.362	9.59	28.077	0.280	0.141	0.228	0.085	0.026	−0.049	-	-	-
5	0.417	0.368	9.56	26.692	0.283	0.145	0.205	0.073	0.023	-	-	-	-
6	0.409	0.370	9.54	26.844	0.284	0.150	0.213	0.074	-	-	-	-	-
7	0.396	0.367	9.56	28.487	0.278	0.164	0.201	-	-	-	-	-	-

**Table A2:** Models obtained through backward regression, considering the nine indicators of the questionnaire as inputs and KPI_Yo-Yo_ as output. In the columns related to the model are reported: model number; R^2^, adjusted R^2^ (R^2^_adj_); and standard error of the estimates (SEE). In the subsequent columns, the constant value for each of the presented models and the BIO_Indices_ with their coefficients are listed. The selected model, in light grey, defines which biometric indicators are most meaningful to estimate KPI_Yo-Yo_. Legend: BMI_Index_ = body mass index; Age_Index_ = age index; PAL_Index_ = physical activity level index; Injury_Index_ = occurring injuries index; Alcohol_Index_ = alcohol consumption index; Work_Index_ = work-related index; Career_Index_ = sports career index; Medical_Index_ = medical history index; Smoke_Index_ = smoke habits index; β = Constant value of the regression model.

Model Statistics for KPI_YoYo_					BIO_indices_								
Model	R^2^	R^2^_adj_	SEE	Β	PAL_Index_	BMI_Index_	Age_Index_	Work_Index_	Career_Index_	Alcohol_Index_	Smoke_Index_	Injury_Index_	Medical_Index_
1	0.559	0.488	13.47	−31.676	0.163	0.447	0.191	0.139	−0.173	0.176	0.202	−0.046	−0.041
2	0.556	0.494	13.39	−35.983	0.164	0.450	0.192	0.142	−0.176	0.175	0.219	−0.058	-
3	0.550	0.496	13.37	−39.479	0.161	0.438	0.198	0.152	−0.193	0.177	0.219	-	-
4	0.539	0.492	13.43	−19.773	0.167	0.438	0.182	0.162	−0.176	0.185	-	-	-
5	0.524	0.484	13.53	−10.477	0.170	0.442	0.196	0.137		-	-	-	-
6	0.509	0.476	13.63	−18.205	0.162	0.449	0.204	0.110	-	-	-	-	-
7	0.497	0.473	13.68	−16.884	0.162	0.438	0.224	-	-	-	-	-	-

**Table A3:** Models obtained through backward regression, considering the nine indicators of the questionnaire as inputs and KPI_SLJ_ as output. In the columns related to the model are reported: model number; R^2^, adjusted R^2^ (R^2^_adj_); and standard error of the estimates (SEE). In the subsequent columns, the constant value for each of the presented models and the BIO_Indices_ with their coefficients are listed. The selected model, in light grey, defines which biometric indicators are most meaningful to estimate KPI_SLJ_. Legend: BMI_Index_ = body mass index; Age_Index_ = age index; PAL_Index_ = physical activity level index; Injury_Index_ = occurring injuries index; Alcohol_Index_ = alcohol consumption index; Work_Index_ = work-related index; Career_Index_ = sports career index; Medical_Index_ = medical history index; Smoke_Index_ = smoke habits index; β = Constant value of the regression model.

Model Statistics for KPI_SLJ_					BIO_indices_								
Model	R^2^	R^2^_adj_	SEE	β	PAL_Index_	BMI_Index_	Age_Index_	Injury_Index_	Career_Index_	Medical_Index_	Smoke_Index_	Work_Index_	Alcohol_Index_
1	0.383	0.283	12.41	36.733	0.066	0.211	0.245	−0.099	0.148	−0.089	0.083	−0.028	0.002
2	0.383	0.296	12.29	36.823	0.066	0.211	0.245	−0.099	0.148	−0.089	0.083	−0.028	-
3	0.382	0.307	12.20	36.975	0.066	0.213	0.240	−0.097	0.142	−0.088	0.079	-	-
4	0.379	0.316	12.12	44.637	0.069	0.213	0.235	−0.096	0.150	−0.093	-	-	-
5	0.356	0.302	12.24	38.323	0.072	0.217	0.237	−0.125	0.148	-	-	-	-

**Table A4:** Models obtained through backward regression, considering the nine indicators of the questionnaire as inputs and KPI_CMJ_ as output. In the columns related to the model are reported: model number; R^2^, adjusted R^2^ (R^2^_adj_); and standard error of the estimates (SEE). In the subsequent columns, the constant value for each of the presented models and the BIO_Indices_ with their coefficients are listed. The selected model, in light grey, defines which biometric indicators are most meaningful to estimate KPI_CMJ_. Legend: BMI_Index_ = body mass index; Age_Index_ = age index; PAL_Index_ = physical activity level index; Injury_Index_ = occurring injuries index; Alcohol_Index_ = alcohol consumption index; Work_Index_ = work-related index; Career_Index_ = sports career index; Medical_Index_ = medical history index; Smoke_Index_ = smoke habits index; β = Constant value of the regression model.

Model Statistics for KPI_CMJ_					BIO_indices_								
Model	R^2^	R^2^_adj_	SEE	β	BMI_Index_	Injury_Index_	Medical_Index_	Age_Index_	Career_Index_	Alcohol_Index_	Smoke_Index_	PAL_Index_	Work_Index_
1	0.446	0.357	11.42	41.768	0.224	−0.190	−0.103	0.224	0.166	0.065	0.084	0.017	−0.043
2	0.443	0.365	11.35	41.468	0.227	−0.187	−0.102	0.216	0.154	0.077	0.084	0.017	-
3	0.440	0.373	11.28	40.370	0.227	−0.183	−0.103	0.220	0.158	0.080	0.094	-	-
4	0.436	0.379	11.22	49.348	0.227	−0.181	−0.110	0.215	0.167	0.082	-	-	-
5	0.431	0.384	11.18	53.438	0.230	−0.181	−0.109	0.219	0.187	-	-	-	-

**Table A5:** Models obtained through backward regression, considering the nine indicators of the questionnaire as inputs and KPI_RSA_ as output. In the columns related to the model are reported: model number; R^2^, adjusted R^2^ (R^2^_adj_); and standard error of the estimates (SEE). In the subsequent columns, the constant value for each of the presented models and the BIO_Indices_ with their coefficients are listed. The selected model, in light grey, defines which biometric indicators are most meaningful to estimate KPI_RSA_. Legend: BMI_Index_ = body mass index; Age_Index_ = age index; PAL_Index_ = physical activity level index; Injury_Index_ = occurring injuries index; Alcohol_Index_ = alcohol consumption index; Work_Index_ = work-related index; Career_Index_ = sports career index; Medical_Index_ = medical history index; Smoke_Index_ = smoke habits index; β = Constant value of the regression model.

Model Statistics for KPI_RSA_					BIO_indices_								
Model	R^2^	R^2^_adj_	SEE	β	BMI_Index_	Medical_Index_	PAL_Index_	Age_Index_	Injury_Index_	Career_Index_	Alcohol_Index_	Work_Index_	Smok_Index_
1	0.198	0.069	31.517	1.973	0.661	0.213	−0.074	0.127	−0.107	0.059	0.098	0.041	−0.042
2	0.198	0.085	31.242	−2.029	0.661	0.216	−0.075	0.130	−0.108	0.056	0.097	0.039	-
3	0.197	0.100	30.981	−1.210	0.659	0.214	−0.075	0.137	−0.111	0.067	0.086	-	-
4	0.196	0.114	30.738	3.048	0.662	0.215	−0.074	0.142	−0.111	0.087	-	-	-
5	0.194	0.126	30.526	7.653	0.653	0.216	−0.069	0.141	−0.100	-	-	-	-
6	0.188	0.135	30.379	3.328	0.635	0.183	−0.077	0.152	-	-	-	-	-
7	0.180	0.140	30.286	12.960	0.672	0.177	−0.071	-	-	-	-	-	-
8	0.170	0.144	30.223	9.548	0.663	0.181	-	-	-	-	-	-	-
9	0.150	0.137	30.338	25.391	0.662	-	-	-	-	-	-	-	-
